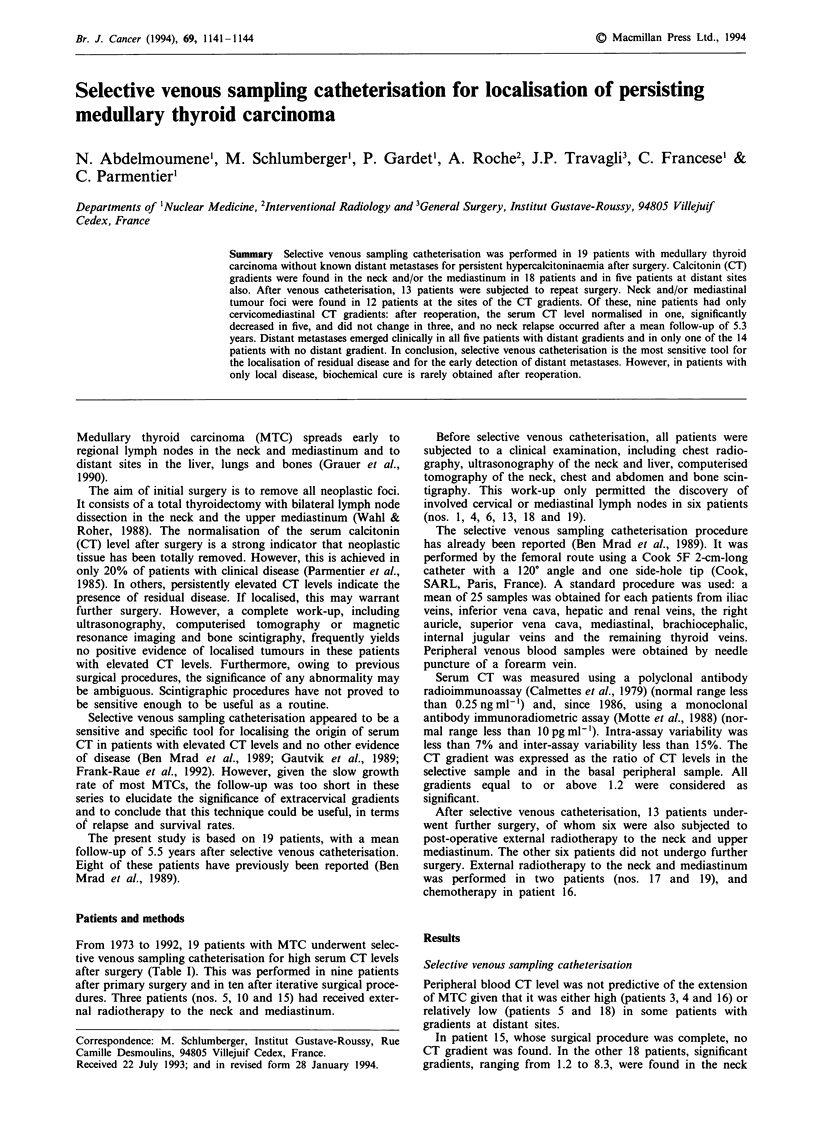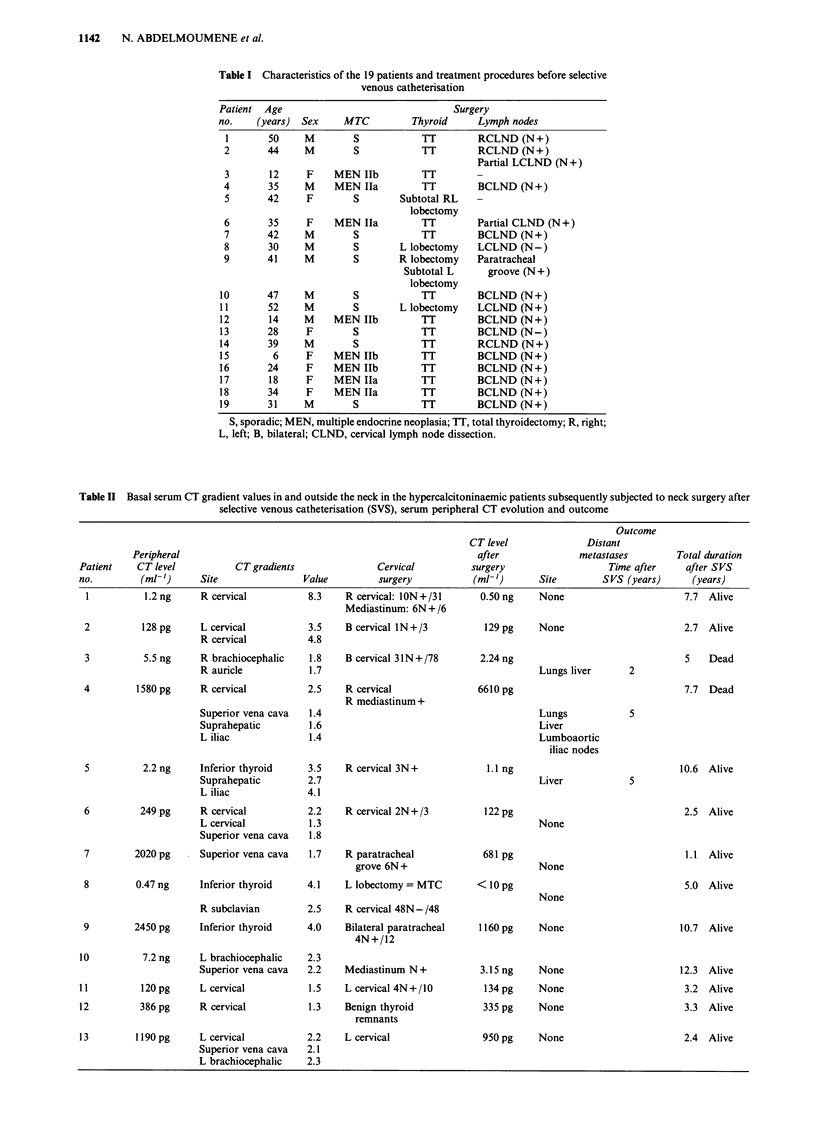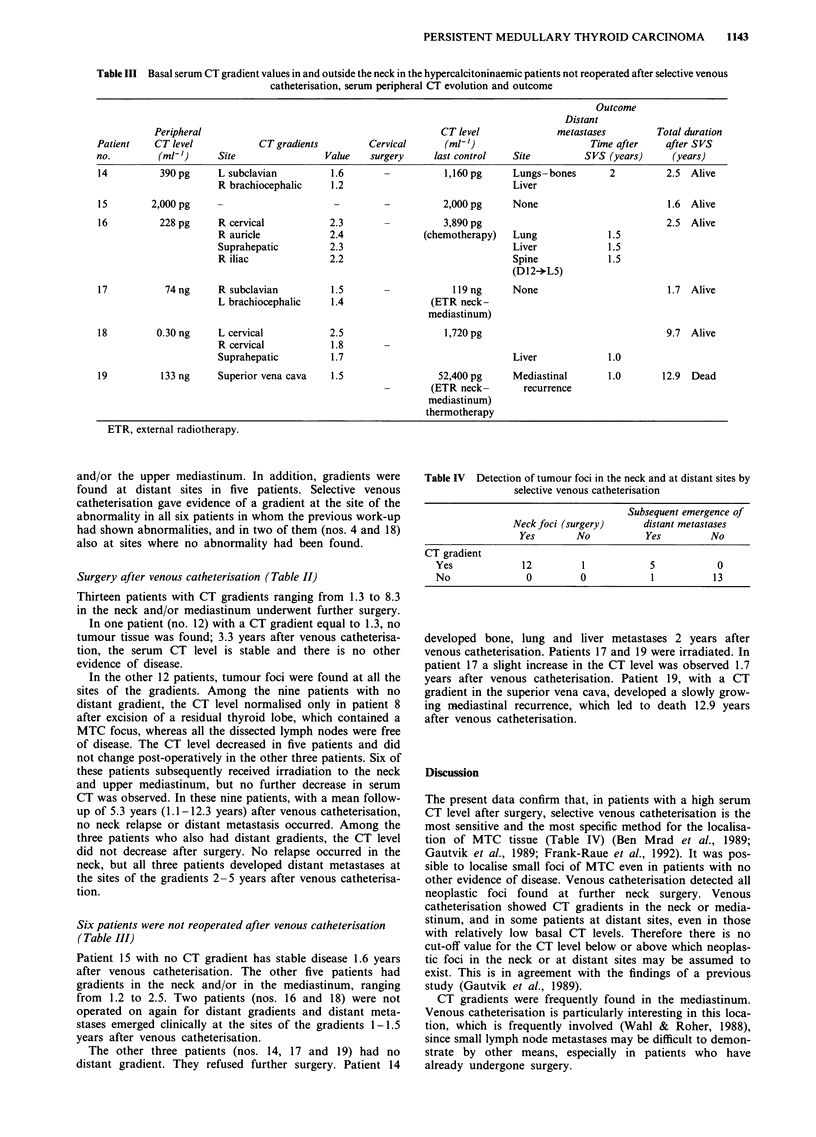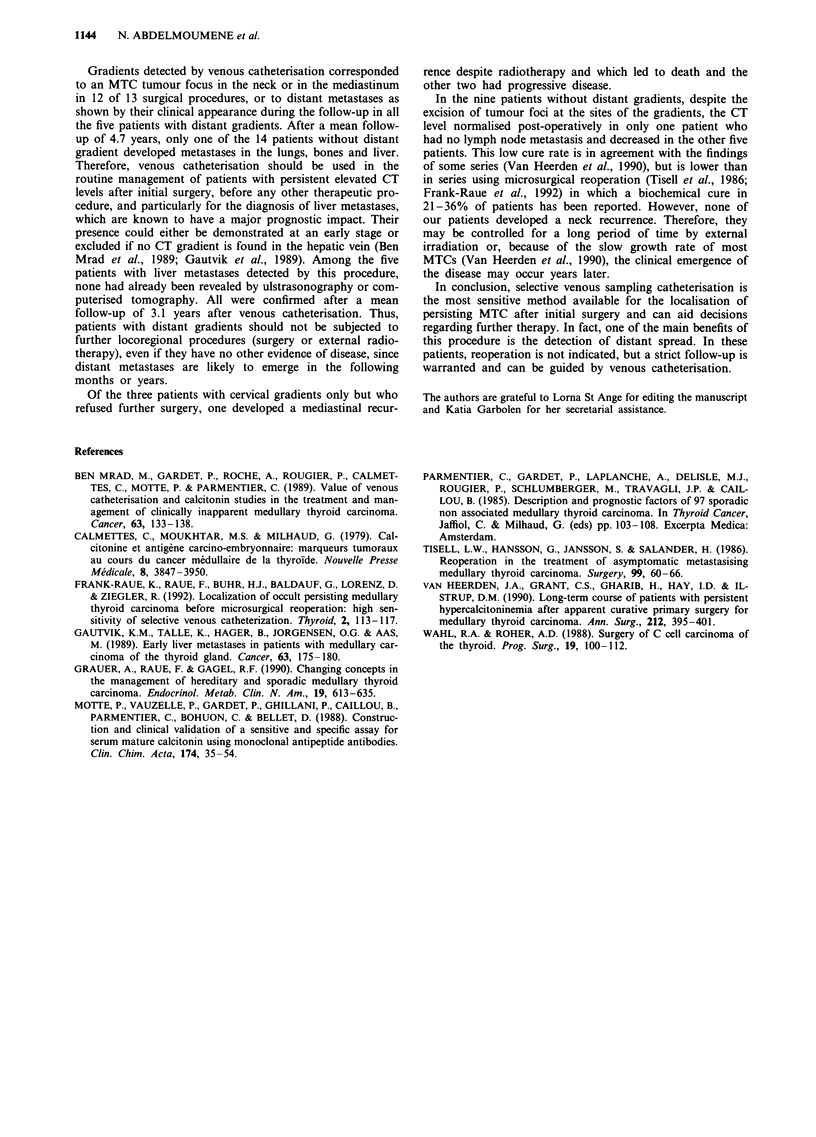# Selective venous sampling catheterisation for localisation of persisting medullary thyroid carcinoma.

**DOI:** 10.1038/bjc.1994.224

**Published:** 1994-06

**Authors:** N. Abdelmoumene, M. Schlumberger, P. Gardet, A. Roche, J. P. Travagli, C. Francese, C. Parmentier

**Affiliations:** Department of Nuclear Medicine, Institut Gustave-Roussy, Villejuif, France.

## Abstract

Selective venous sampling catheterisation was performed in 19 patients with medullary thyroid carcinoma without known distant metastases for persistent hypercalcitoninaemia after surgery. Calcitonin (CT) gradients were found in the neck and/or the mediastinum in 18 patients and in five patients at distant sites also. After venous catheterisation, 13 patients were subjected to repeat surgery. Neck and/or mediastinal tumour foci were found in 12 patients at the sites of the CT gradients. Of these, nine patients had only cervicomediastinal CT gradients: after reoperation, the serum CT level normalised in one, significantly decreased in five, and did not change in three, and no neck relapse occurred after a mean follow-up of 5.3 years. Distant metastases emerged clinically in all five patients with distant gradients and in only one of the 14 patients with no distant gradient. In conclusion, selective venous catheterisation is the most sensitive tool for the localisation of residual disease and for the early detection of distant metastases. However, in patients with only local disease, biochemical cure is rarely obtained after reoperation.


Br. J. Cancer (1994), 69, 1141–1144 © Macmillan Press Ltd., 1994

### Selective venous sampling catheterisation for localisation of persisting medullary thyroid carcinoma

N. Abdelmoumene[1], M. Schlumberger[1], P. Gardet[1], A. Roche[2], J.P. Travagli[3], C. Francese[1] & C. Parmentier[1]

*Departments of [1]Nuclear Medicine, [2]Interventional Radiology and [3]General Surgery, Institut Gustave-Roussy, 94805 Villejuif Cedex, France*

**Summary** Selective venous sampling catheterisation was performed in 19 patients with medullary thyroid carcinoma without known distant metastases for persistent hypercalcitoninaemia after surgery. Calcitonin (CT) gradients were found in the neck and/or the mediastinum in 18 patients and in five patients at distant sites also. After venous catheterisation, 13 patients were subjected to repeat surgery. Neck and/or mediastinal tumour foci were found in 12 patients at the sites of the CT gradients. Of these, nine patients had only cervicomediastinal CT gradients: after reoperation, the serum CT level normalised in one, significantly decreased in five, and did not change in three, and no neck relapse occurred after a mean follow-up of 5.3 years. Distant metastases emerged clinically in all five patients with distant gradients and in only one of the 14 patients with no distant gradient. In conclusion, selective venous catheterisation is the most sensitive tool for the localisation of residual disease and for the early detection of distant metastases. However, in patients with only local disease, biochemical cure is rarely obtained after reoperation.

Medullary thyroid carcinoma (MTC) spreads early to regional lymph nodes in the neck and mediastinum and to distant sites in the liver, lungs and bones (Grauer *et al.*, 1990).

The aim of initial surgery is to remove all neoplastic foci. It consists of a total thyroidectomy with bilateral lymph node dissection in the neck and the upper mediastinum (Wahl & Roher, 1988). The normalisation of the serum calcitonin (CT) level after surgery is a strong indicator that neoplastic tissue has been totally removed. However, this is achieved in only 20% of patients with clinical disease (Parmentier *et al.*, 1985). In others, persistently elevated CT levels indicate the presence of residual disease. If localised, this may warrant further surgery. However, a complete work-up, including ultrasonography, computerised tomography or magnetic resonance imaging and bone scintigraphy, frequently yields no positive evidence of localised tumours in these patients with elevated CT levels. Furthermore, owing to previous surgical procedures, the significance of any abnormality may be ambiguous. Scintigraphic procedures have not proved to be sensitive enough to be useful as a routine.

Selective venous sampling catheterisation appeared to be a sensitive and specific tool for localising the origin of serum CT in patients with elevated CT levels and no other evidence of disease (Ben Mrad *et al.*, 1989; Gautvik *et al.*, 1989; Frank-Raue *et al.*, 1992). However, given the slow growth rate of most MTCs, the follow-up was too short in these series to elucidate the significance of extracervical gradients and to conclude that this technique could be useful, in terms of relapse and survival rates.

The present study is based on 19 patients, with a mean follow-up of 5.5 years after selective venous catheterisation. Eight of these patients have previously been reported (Ben Mrad *et al.*, 1989).

### Patients and methods

From 1973 to 1992, 19 patients with MTC underwent selective venous sampling catheterisation for high serum CT levels after surgery (Table I). This was performed in nine patients after primary surgery and in ten after iterative surgical procedures. Three patients (nos. 5, 10 and 15) had received external radiotherapy to the neck and mediastinum.

Correspondence: M. Schlumberger, Institut Gustave-Roussy, Rue Camille Desmoulins, 94805 Villejuif Cedex, France.
Received 22 July 1993; and in revised form 28 January 1994.

Before selective venous catheterisation, all patients were subjected to a clinical examination, including chest radiography, ultrasonography of the neck and liver, computerised tomography of the neck, chest and abdomen and bone scintigraphy. This work-up only permitted the discovery of involved cervical or mediastinal lymph nodes in six patients (nos. 1, 4, 6, 13, 18 and 19).

The selective venous sampling catheterisation procedure has already been reported (Ben Mrad *et al.*, 1989). It was performed by the femoral route using a Cook 5F 2-cm-long catheter with a 120° angle and one side-hole tip (Cook, SARL, Paris, France). A standard procedure was used: a mean of 25 samples was obtained for each patients from iliac veins, inferior vena cava, hepatic and renal veins, the right auricle, superior vena cava, mediastinal, brachiocephalic, internal jugular veins and the remaining thyroid veins. Peripheral venous blood samples were obtained by needle puncture of a forearm vein.

Serum CT was measured using a polyclonal antibody radioimmunoassay (Calmettes *et al.*, 1979) (normal range less than $0.25\,ng\,ml^{-1}$) and, since 1986, using a monoclonal antibody immunoradiometric assay (Motte *et al.*, 1988) (normal range less than $10\,pg\,ml^{-1}$). Intra-assay variability was less than 7% and inter-assay variability less than 15%. The CT gradient was expressed as the ratio of CT levels in the selective sample and in the basal peripheral sample. All gradients equal to or above 1.2 were considered as significant.

After selective venous catheterisation, 13 patients underwent further surgery, of whom six were also subjected to post-operative external radiotherapy to the neck and upper mediastinum. The other six patients did not undergo further surgery. External radiotherapy to the neck and mediastinum was performed in two patients (nos. 17 and 19), and chemotherapy in patient 16.

### Results

### *Selective venous sampling catheterisation*

Peripheral blood CT level was not predictive of the extension of MTC given that it was either high (patients 3, 4 and 16) or relatively low (patients 5 and 18) in some patients with gradients at distant sites.

In patient 15, whose surgical procedure was complete, no CT gradient was found. In the other 18 patients, significant gradients, ranging from 1.2 to 8.3, were found in the neck

**Table I** Characteristics of the 19 patients and treatment procedures before selective venous catheterisation

| *Patient no.* | *Age (years)* | *Sex* | *MTC* | *Surgery* *Thyroid* | *Lymph nodes* |
|---|---|---|---|---|---|
| 1 | 50 | M | S | TT | RCLND (N+) |
| 2 | 44 | M | S | TT | RCLND (N+) |
| | | | | | Partial LCLND (N+) |
| 3 | 12 | F | MEN IIb | TT | – |
| 4 | 35 | M | MEN IIa | TT | BCLND (N+) |
| 5 | 42 | F | S | Subtotal RL lobectomy | – |
| 6 | 35 | F | MEN IIa | TT | Partial CLND (N+) |
| 7 | 42 | M | S | TT | BCLND (N+) |
| 8 | 30 | M | S | L lobectomy | LCLND (N–) |
| 9 | 41 | M | S | R lobectomy | Paratracheal groove (N+) |
| | | | | Subtotal L lobectomy | |
| 10 | 47 | M | S | TT | BCLND (N+) |
| 11 | 52 | M | S | L lobectomy | LCLND (N+) |
| 12 | 14 | M | MEN IIb | TT | BCLND (N+) |
| 13 | 28 | F | S | TT | BCLND (N–) |
| 14 | 39 | M | S | TT | RCLND (N+) |
| 15 | 6 | F | MEN IIb | TT | BCLND (N+) |
| 16 | 24 | F | MEN IIb | TT | BCLND (N+) |
| 17 | 18 | F | MEN IIa | TT | BCLND (N+) |
| 18 | 34 | F | MEN IIa | TT | BCLND (N+) |
| 19 | 31 | M | S | TT | BCLND (N+) |

S, sporadic; MEN, multiple endocrine neoplasia; TT, total thyroidectomy; R, right; L, left; B, bilateral; CLND, cervical lymph node dissection.

**Table II** Basal serum CT gradient values in and outside the neck in the hypercalcitoninaemic patients subsequently subjected to neck surgery after selective venous catheterisation (SVS), serum peripheral CT evolution and outcome

| *Patient no.* | *Peripheral CT level ($ml^{-1}$)* | *CT gradients Site* | *Value* | *Cervical surgery* | *CT level after surgery ($ml^{-1}$)* | *Outcome Distant metastases Site* | *Time after SVS (years)* | *Total duration after SVS (years)* | |
|---|---|---|---|---|---|---|---|---|---|
| 1 | 1.2 ng | R cervical | 8.3 | R cervical: 10N+/31 | 0.50 ng | None | | 7.7 | Alive |
| | | | | Mediastinum: 6N+/6 | | | | | |
| 2 | 128 pg | L cervical | 3.5 | B cervical 1N+/3 | 129 pg | None | | 2.7 | Alive |
| | | R cervical | 4.8 | | | | | | |
| 3 | 5.5 ng | R brachiocephalic | 1.8 | B cervical 31N+/78 | 2.24 ng | | | 5 | Dead |
| | | R auricle | 1.7 | | | Lungs liver | 2 | | |
| 4 | 1580 pg | R cervical | 2.5 | R cervical | 6610 pg | | | 7.7 | Dead |
| | | | | R mediastinum+ | | | | | |
| | | Superior vena cava | 1.4 | | | Lungs | 5 | | |
| | | Suprahepatic | 1.6 | | | Liver | | | |
| | | L iliac | 1.4 | | | Lumboaortic iliac nodes | | | |
| 5 | 2.2 ng | Inferior thyroid | 3.5 | R cervical 3N+ | 1.1 ng | | | 10.6 | Alive |
| | | Suprahepatic | 2.7 | | | Liver | 5 | | |
| | | L iliac | 4.1 | | | | | | |
| 6 | 249 pg | R cervical | 2.2 | R cervical 2N+/3 | 122 pg | | | 2.5 | Alive |
| | | L cervical | 1.3 | | | None | | | |
| | | Superior vena cava | 1.8 | | | | | | |
| 7 | 2020 pg | Superior vena cava | 1.7 | R paratracheal grove 6N+ | 681 pg | None | | 1.1 | Alive |
| 8 | 0.47 ng | Inferior thyroid | 4.1 | L lobectomy = MTC | <10 pg | None | | 5.0 | Alive |
| | | R subclavian | 2.5 | R cervical 48N–/48 | | | | | |
| 9 | 2450 pg | Inferior thyroid | 4.0 | Bilateral paratracheal 4N+/12 | 1160 pg | None | | 10.7 | Alive |
| 10 | 7.2 ng | L brachiocephalic | 2.3 | | | | | | |
| | | Superior vena cava | 2.2 | Mediastinum N+ | 3.15 ng | None | | 12.3 | Alive |
| 11 | 120 pg | L cervical | 1.5 | L cervical 4N+/10 | 134 pg | None | | 3.2 | Alive |
| 12 | 386 pg | R cervical | 1.3 | Benign thyroid remnants | 335 pg | None | | 3.3 | Alive |
| 13 | 1190 pg | L cervical | 2.2 | L cervical | 950 pg | None | | 2.4 | Alive |
| | | Superior vena cava | 2.1 | | | | | | |
| | | L brachiocephalic | 2.3 | | | | | | |

**Table III** Basal serum CT gradient values in and outside the neck in the hypercalcitoninaemic patients not reoperated after selective venous catheterisation, serum peripheral CT evolution and outcome

| Patient no. | Peripheral CT level ($ml^{-1}$) | CT gradients Site | CT gradients Value | Cervical surgery | CT level ($ml^{-1}$) last control | Outcome: Distant metastases Site | Outcome: Distant metastases Time after SVS (years) | Total duration after SVS (years) | |
|---|---|---|---|---|---|---|---|---|---|
| 14 | 390 pg | L subclavian | 1.6 | – | 1,160 pg | Lungs–bones | 2 | 2.5 | Alive |
| | | R brachiocephalic | 1.2 | | | Liver | | | |
| 15 | 2,000 pg | – | – | – | 2,000 pg | None | | 1.6 | Alive |
| 16 | 228 pg | R cervical | 2.3 | – | 3,890 pg | | | 2.5 | Alive |
| | | R auricle | 2.4 | | (chemotherapy) | Lung | 1.5 | | |
| | | Suprahepatic | 2.3 | | | Liver | 1.5 | | |
| | | R iliac | 2.2 | | | Spine (D12→L5) | 1.5 | | |
| 17 | 74 ng | R subclavian | 1.5 | – | 119 ng | None | | 1.7 | Alive |
| | | L brachiocephalic | 1.4 | | (ETR neck–mediastinum) | | | | |
| 18 | 0.30 ng | L cervical | 2.5 | | 1,720 pg | | | 9.7 | Alive |
| | | R cervical | 1.8 | – | | | | | |
| | | Suprahepatic | 1.7 | | | Liver | 1.0 | | |
| 19 | 133 ng | Superior vena cava | 1.5 | | 52,400 pg | Mediastinal recurrence | 1.0 | 12.9 | Dead |
| | | | | – | (ETR neck–mediastinum) thermotherapy | | | | |

ETR, external radiotherapy.

and/or the upper mediastinum. In addition, gradients were found at distant sites in five patients. Selective venous catheterisation gave evidence of a gradient at the site of the abnormality in all six patients in whom the previous work-up had shown abnormalities, and in two of them (nos. 4 and 18) also at sites where no abnormality had been found.

*Surgery after venous catheterisation (Table II)*

Thirteen patients with CT gradients ranging from 1.3 to 8.3 in the neck and/or mediastinum underwent further surgery.

In one patient (no. 12) with a CT gradient equal to 1.3, no tumour tissue was found; 3.3 years after venous catheterisation, the serum CT level is stable and there is no other evidence of disease.

In the other 12 patients, tumour foci were found at all the sites of the gradients. Among the nine patients with no distant gradient, the CT level normalised only in patient 8 after excision of a residual thyroid lobe, which contained a MTC focus, whereas all the dissected lymph nodes were free of disease. The CT level decreased in five patients and did not change post-operatively in the other three patients. Six of these patients subsequently received irradiation to the neck and upper mediastinum, but no further decrease in serum CT was observed. In these nine patients, with a mean follow-up of 5.3 years (1.1–12.3 years) after venous catheterisation, no neck relapse or distant metastasis occurred. Among the three patients who also had distant gradients, the CT level did not decrease after surgery. No relapse occurred in the neck, but all three patients developed distant metastases at the sites of the gradients 2–5 years after venous catheterisation.

*Six patients were not reoperated after venous catheterisation (Table III)*

Patient 15 with no CT gradient has stable disease 1.6 years after venous catheterisation. The other five patients had gradients in the neck and/or in the mediastinum, ranging from 1.2 to 2.5. Two patients (nos. 16 and 18) were not operated on again for distant gradients and distant metastases emerged clinically at the sites of the gradients 1–1.5 years after venous catheterisation.

The other three patients (nos. 14, 17 and 19) had no distant gradient. They refused further surgery. Patient 14 developed bone, lung and liver metastases 2 years after venous catheterisation. Patients 17 and 19 were irradiated. In patient 17 a slight increase in the CT level was observed 1.7 years after venous catheterisation. Patient 19, with a CT gradient in the superior vena cava, developed a slowly growing mediastinal recurrence, which led to death 12.9 years after venous catheterisation.

**Table IV** Detection of tumour foci in the neck and at distant sites by selective venous catheterisation

| | Neck foci (surgery) Yes | Neck foci (surgery) No | Subsequent emergence of distant metastases Yes | Subsequent emergence of distant metastases No |
|---|---|---|---|---|
| CT gradient | | | | |
| Yes | 12 | 1 | 5 | 0 |
| No | 0 | 0 | 1 | 13 |

### Discussion

The present data confirm that, in patients with a high serum CT level after surgery, selective venous catheterisation is the most sensitive and the most specific method for the localisation of MTC tissue (Table IV) (Ben Mrad *et al.*, 1989; Gautvik *et al.*, 1989; Frank-Raue *et al.*, 1992). It was possible to localise small foci of MTC even in patients with no other evidence of disease. Venous catheterisation detected all neoplastic foci found at further neck surgery. Venous catheterisation showed CT gradients in the neck or mediastinum, and in some patients at distant sites, even in those with relatively low basal CT levels. Therefore there is no cut-off value for the CT level below or above which neoplastic foci in the neck or at distant sites may be assumed to exist. This is in agreement with the findings of a previous study (Gautvik *et al.*, 1989).

CT gradients were frequently found in the mediastinum. Venous catheterisation is particularly interesting in this location, which is frequently involved (Wahl & Roher, 1988), since small lymph node metastases may be difficult to demonstrate by other means, especially in patients who have already undergone surgery.

Gradients detected by venous catheterisation corresponded to an MTC tumour focus in the neck or in the mediastinum in 12 of 13 surgical procedures, or to distant metastases as shown by their clinical appearance during the follow-up in all the five patients with distant gradients. After a mean follow-up of 4.7 years, only one of the 14 patients without distant gradient developed metastases in the lungs, bones and liver. Therefore, venous catheterisation should be used in the routine management of patients with persistent elevated CT levels after initial surgery, before any other therapeutic procedure, and particularly for the diagnosis of liver metastases, which are known to have a major prognostic impact. Their presence could either be demonstrated at an early stage or excluded if no CT gradient is found in the hepatic vein (Ben Mrad *et al.*, 1989; Gautvik *et al.*, 1989). Among the five patients with liver metastases detected by this procedure, none had already been revealed by ulstrasonography or computerised tomography. All were confirmed after a mean follow-up of 3.1 years after venous catheterisation. Thus, patients with distant gradients should not be subjected to further locoregional procedures (surgery or external radiotherapy), even if they have no other evidence of disease, since distant metastases are likely to emerge in the following months or years.

Of the three patients with cervical gradients only but who refused further surgery, one developed a mediastinal recurrence despite radiotherapy and which led to death and the other two had progressive disease.

In the nine patients without distant gradients, despite the excision of tumour foci at the sites of the gradients, the CT level normalised post-operatively in only one patient who had no lymph node metastasis and decreased in the other five patients. This low cure rate is in agreement with the findings of some series (Van Heerden *et al.*, 1990), but is lower than in series using microsurgical reoperation (Tisell *et al.*, 1986; Frank-Raue *et al.*, 1992) in which a biochemical cure in 21–36% of patients has been reported. However, none of our patients developed a neck recurrence. Therefore, they may be controlled for a long period of time by external irradiation or, because of the slow growth rate of most MTCs (Van Heerden *et al.*, 1990), the clinical emergence of the disease may occur years later.

In conclusion, selective venous sampling catheterisation is the most sensitive method available for the localisation of persisting MTC after initial surgery and can aid decisions regarding further therapy. In fact, one of the main benefits of this procedure is the detection of distant spread. In these patients, reoperation is not indicated, but a strict follow-up is warranted and can be guided by venous catheterisation.

The authors are grateful to Lorna St Ange for editing the manuscript and Katia Garbolen for her secretarial assistance.

### References

BEN MRAD, M., GARDET, P., ROCHE, A., ROUGIER, P., CALMETTES, C., MOTTE, P. & PARMENTIER, C. (1989). Value of venous catheterisation and calcitonin studies in the treatment and management of clinically inapparent medullary thyroid carcinoma. *Cancer*, **63**, 133–138.

CALMETTES, C., MOUKHTAR, M.S. & MILHAUD, G. (1979). Calcitonine et antigène carcino-embryonnaire: marqueurs tumoraux au cours du cancer médullaire de la thyroïde. *Nouvelle Presse Médicale*, **8**, 3847–3950.

FRANK-RAUE, K., RAUE, F., BUHR, H.J., BALDAUF, G., LORENZ, D. & ZIEGLER, R. (1992). Localization of occult persisting medullary thyroid carcinoma before microsurgical reoperation: high sensitivity of selective venous catheterization. *Thyroid*, **2**, 113–117.

GAUTVIK, K.M., TALLE, K., HAGER, B., JORGENSEN, O.G. & AAS, M. (1989). Early liver metastases in patients with medullary carcinoma of the thyroid gland. *Cancer*, **63**, 175–180.

GRAUER, A., RAUE, F. & GAGEL, R.F. (1990). Changing concepts in the management of hereditary and sporadic medullary thyroid carcinoma. *Endocrinol. Metab. Clin. N. Am.*, **19**, 613–635.

MOTTE, P., VAUZELLE, P., GARDET, P., GHILLANI, P., CAILLOU, B., PARMENTIER, C., BOHUON, C. & BELLET, D. (1988). Construction and clinical validation of a sensitive and specific assay for serum mature calcitonin using monoclonal antipeptide antibodies. *Clin. Chim. Acta*, **174**, 35–54.

PARMENTIER, C., GARDET, P., LAPLANCHE, A., DELISLE, M.J., ROUGIER, P., SCHLUMBERGER, M., TRAVAGLI, J.P. & CAILLOU, B. (1985). Description and prognostic factors of 97 sporadic non associated medullary thyroid carcinoma. In *Thyroid Cancer*, Jaffiol, C. & Milhaud, G. (eds) pp. 103–108. Excerpta Medica: Amsterdam.

TISELL, L.W., HANSSON, G., JANSSON, S. & SALANDER, H. (1986). Reoperation in the treatment of asymptomatic metastasising medullary thyroid carcinoma. *Surgery*, **99**, 60–66.

VAN HEERDEN, J.A., GRANT, C.S., GHARIB, H., HAY, I.D. & ILSTRUP, D.M. (1990). Long-term course of patients with persistent hypercalcitoninemia after apparent curative primary surgery for medullary thyroid carcinoma. *Ann. Surg.*, **212**, 395–401.

WAHL, R.A. & ROHER, A.D. (1988). Surgery of C cell carcinoma of the thyroid. *Prog. Surg.*, **19**, 100–112.